# ESTs and EST-linked polymorphisms for genetic mapping and phylogenetic reconstruction in the guppy, *Poecilia reticulata*

**DOI:** 10.1186/1471-2164-8-269

**Published:** 2007-08-08

**Authors:** Christine Dreyer, Margarete Hoffmann, Christa Lanz, Eva-Maria Willing, Markus Riester, Norman Warthmann, Andrea Sprecher, Namita Tripathi, Stefan R Henz, Detlef Weigel

**Affiliations:** 1 Department of Molecular Biology, Max Planck Institute for Developmental Biology, Tübingen, D72076, Germany

## Abstract

**Background:**

The guppy, *Poecilia reticulata*, is a well-known model organism for studying inheritance and variation of male ornamental traits as well as adaptation to different river habitats. However, genomic resources for studying this important model were not previously widely available.

**Results:**

With the aim of generating molecular markers for genetic mapping of the guppy, cDNA libraries were constructed from embryos and different adult organs to generate expressed sequence tags (ESTs). About 18,000 ESTs were annotated according to BLASTN and BLASTX results and the sequence information from the 3' UTRs was exploited to generate PCR primers for re-sequencing of genomic DNA from different wild type strains. By comparison of EST-linked genomic sequences from at least four different ecotypes, about 1,700 polymorphisms were identified, representing about 400 distinct genes. Two interconnected MySQL databases were built to organize the ESTs and markers, respectively. A robust phylogeny of the guppy was reconstructed, based on 10 different nuclear genes.

**Conclusion:**

Our EST and marker databases provide useful tools for genetic mapping and phylogenetic studies of the guppy.

## Background

The Trinidadian guppy, *Poecilia reticulata *Peters, is well known for the highly polymorphic male color patterns, which have been the subject of genetic analysis for almost a century [1]. The vast literature on the ecology and evolution of the guppy and the extensive phenotypic variation in wild populations make the guppy a particularly attractive choice for understanding the molecular basis of adaptation to varying natural conditions. Despite the wealth of field studies, molecular genetic information about the guppy is still scarce. Therefore, a genetic map would be a first step towards identifying quantitative adaptive traits of the guppy, including male ornamentation and predator-driven adaptations found in different river habitats in Trinidad, underlying heritable differences in life history traits [2,3].

The genome size of the guppy is estimated to be around 740 Mbp, with a diploid set of 46 chromosomes, including genetically defined X and Y sex chromosomes [4]. A first map of the sex chromosomes, based on classical genetic analysis of male colour patterns, has been sketched out by Winge and co-workers [5]. More recently, Phang and co-workers used ornamental guppies from Singapore to generate a genetic map based on 300 RAPD markers, and a cross between two laboratory strains of different body shape and colour was mapped using a combination of 186 AFLP and microsatellite markers [6-8]. Conservation of microsatellites between closely related species and synteny with respect to 61 microsatellite markers were suggested by an intergeneric cross between *Xiphophorus maculatus *[9] and *P. reticulata *[10]. In all of these studies, the number of linkage groups fell short of the chromosome number, indicating that a higher marker density is required for complete coverage of the genetic map. Unfortunately, RAPD and AFLP markers cannot be easily reused for studying crosses between outbred strains of wild guppies.

We have identified hundreds of expressed sequence tag (EST)-linked single nucleotide polymorphism (SNP) markers, suitable for genetic mapping of wild guppies. The fact that these markers are linked to expressed genes will help to exploit syntenic information from fully sequenced genomes of other fish species [11]. This will in turn also facilitate future identification of candidate genes when mapping qualitative morphological as well as quantitative life history traits of the guppy.

## Results and discussion

### A *P. reticulata *EST database

Several guppy cDNA libraries were constructed using SMART technology, as detailed in Materials and Methods. As sources of mRNA we used whole embryos, newborn fish, adult liver, testis, brain, retina, and skin, in order to obtain a broad spectrum of different expressed sequences. Several feral and laboratory strains were used, including the Quare6 strain from East Trinidad and the Tranquille strain from West Trinidad [12]. This allows for direct sequence comparison of abundant transcripts between different strains. Between 100 and 5,700 clones were picked at random from each library, depending on its complexity. The inserts were first sequenced from the 5' end and sequences were compared to EMBL vertebrate databases (see Methods) using NCBI BLASTN and BLASTX algorithm [13] to assign a possible function. BLAST results were parsed and automatically entered into a MySQL EST database [14]. Sequences lacking sufficiently good support by BLAST hits with an e-value higher than 10^-5 ^were not entered into the database, but were set aside for periodically repeated subsequent BLAST searches.

Our EST database http://guppy.weigelworld.org currently comprises about 18,000 entries, which represent about 5,300 different gene products. The Quare and Tranquille strains are represented by about 40% of the entries each, with the remaining 20% derived from other strains, including the laboratory strains Blue and Istanbul wild [15] (Table 1). About 16,200 ESTs with a minimum of 200 bp sequence available have been deposited to Genbank. The accession numbers are included in Table 1.

**Table 1:** Origin of guppy ESTs

**Source of cDNA**	Clones*	**Strain**	**Clones**
Tranquille Embryo	4885		
Tranquille Liver	2007	Tranquille	6892
Quare Embryo	2731		
Quare Testis	1536		
Quare Head	143		
Quare Liver	554	Quare	4964
Oropuche Skin	1483		
Oropuche Retina	837		
Oropuche Embryo	84	Oropuche	2404
Blue Testis	2560		
Blue Brain	951	Blue	3511
Istanbul wild Skin	348	Istanbul wild	348

Total	18119		18119

*Clones for which sequence information was less than 200 nucleotides have not yet been submitted to Genbank. The accession numbers of the submitted clones are: ES370951–ES387146

**Figure 1:**
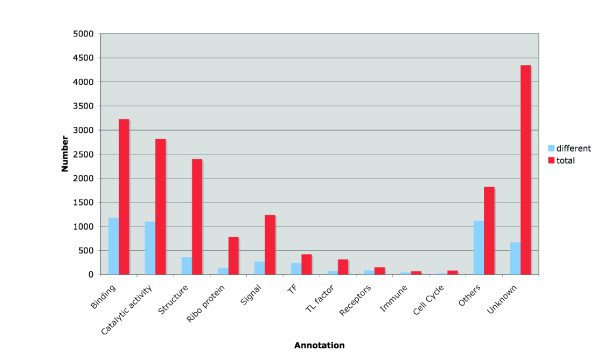
**Classification of deduced gene products represented in the guppy EST library**. Annotations of the best hits found by BLASTX searches against public databases were parsed and classified according to molecular function or biological process. The group of others includes about 3000 ESTs with hits whose E-value was not better than 10^-5 ^Red: number of different ESTs, blue: number of total ESTs.

The results of the BLASTX searches were parsed for annotation according to GO criteria [16], describing molecular function or biological processes, provided that the E-value of the best hit was lower than 10^-5 ^(Fig. 1). Most of the proteins encoded by non-redundant annotated guppy ESTs have binding (17%) or catalytic (13%) activity or are structural proteins (13%). This distribution resembles that described for the *Fundulus heteroclitus *EST database [17], although the guppy database contains relatively more ESTs derived from retina, testis, and muscle. Among enzymes and structural proteins, muscle proteins are the most abundant, because somite tissue prevails in advanced embryos, the predominant source of our EST libraries (Table 1). Sequences without significant best BLAST hits or with hits to genes that lacked informative annotation, e.g. from genomic surveys or non-annotated EST products, are listed as unknown in Table 2. About 25% of the guppy cDNAs in our database is annotated as unknown.

**Table 2:** Composition of guppy EST database by annotation

GO criterion	Different	Total	Most frequent	Number	% In this category
Binding	1175	3229	Apolipoprotein A-IV-4	256	7.9
Catalytic activity	1098	2815	Muscle CKM1	271	9.2
Structure	357	2396	MLC-2	278	11.6
Ribosomal protein	135	783	L3	88	11.2
Signal transduction	271	1235	DEAD box DDX5	478	38.7
Transcription factor	236	425	Cold shock domain fruYP1	43	11.1
Translation factor	71	314	EF1a	149	47.5
Receptors	89	152	Receptor for activated prot K	15	9.9
Immune system	43	69	CD6 precursor	6	0.9
Cell cycle	29	84	Cyclin G1	18	21.4
Proteolysis	9	12	20s proteasome	5	41.7
Others	1108	1809	Vitellogenin	51	28.2
Unknown	670	4346	No significant homology	3000	69.0
SUM	5291	17669			

The EST database can be searched by clone name, by accession number of the best BLAST search hit, and by possible biological function of the deduced protein product of each EST. Furthermore, the BLAST hits are saved in a table, in which a full text search for annotations as well as accession numbers can be performed. A subset of EST clones was also sequenced from the 3' end, and the longest ORF was extracted from the assembly with its 5' sequence. Information on the coding regions was obtained by database searches using BLASTX. Contigs representing multiple hits of the same gene product were aligned in order to analyse these for polymorphisms.

We provide a download function for the extraction of the 5' and 3' end cDNA sequences, as well as assemblies in the FASTA format [18]. It is also possible to perform BLAST searches against all ESTs.

### Development of single nucleotide polymorphism (SNP) markers in guppies

For identification of polymorphisms, we used ESTs to design PCR primers for amplification from genomic DNA of different strains. We primarily amplified 3' UTR sequences, because they are less conserved than coding sequences and not interrupted by introns. The 3' UTRs are typically shorter than in mammals, as has been reported for other lower vertebrates [19]. This somewhat reduces the usefulness of 3' UTRs for identification of SNPs by resequencing of genomic DNA from different strains. Several EST libraries were constructed (Table 1) and some consisted of size-selected sub fractions (data not shown). In the different libraries, between 10 and 30 % of the cDNA sequences had 3' UTRs longer than 400 bp and those were given priority. The 3' end of the coding sequence was included where required to produce fragments about 400 to 500 bp in length. In many instances, PCR amplification and sequencing revealed that the 3' ends of coding regions contained short (< 100 bp) introns. A comparison of ESTs from different strains reveals that, as expected, coding sequences are less polymorphic than the 3' UTRs.

Some primers that were designed to flank an intron were also used, yet their efficient design required prediction of the most likely exon-intron boundaries by alignment of the ESTs to genomic sequences from other fish species, and the length of the resulting PCR products was unpredictable. Aside from SNPs, 3' UTRs and introns also contained short insertions and deletions (indels) at lower frequency (about 20% of all polymorphisms). Of these indels, about 60 % were found as parts of either short tandem repeat polymorphisms or of homopolymer stretches (data not shown).

A second MySQL database was established for the management of the strain-specific markers and was linked to the EST database. From the marker database information can be retrieved on the reference clone, the primer pairs used, type (SNP, indel), and position of polymorphisms between genomic sequences of the different guppy strains. The marker database can also accommodate information on available assays for these polymorphisms. So far, these are mainly MALDI TOF assays developed for high throughput detection of SNP markers [20]. All multiple alignments of genomic and cDNA sequences that had been generated for polymorphism detection [21] were loaded into the multiple SNP query tool (MSQT), a database used for SNP assay development, especially for design of strain-specific assays (Warthmann, Fitz and Weigel, submitted).

From 400 unique ESTs that were successfully re-sequenced in 16 strains of guppy, we could identify 1,700 EST linked polymorphic loci. About 75% of the polymorphisms were identified by comparison of only four populations, originally collected in the Quare (East Trinidad), Tranquille and Upper Aripo rivers (West Trinidad), and in Central Cumaná (Venezuela). The geographically most distant among these are the Quare and the Cumaná guppies and we found these to be most genetically divergent. As shown in Table 3, the evaluation of 400 polymorphic ESTs using the MSQT, allowed for the selection of 235 assays for markers that are each linked to a different gene product and that distinguish between Quare and Cumaná populations. A similar or slightly smaller number of assays were predicted to distinguish other pairs of ecotypes (Table 3). For analysis of genetic crosses between different feral guppy strains, these EST-linked SNP markers can be supplemented by about 50 microsatellite markers previously described in guppies [10,23,24].

**Table 3:** Polymorphisms in nuclear genes that distinguish strains

Strain	Quare	Cumaná [24]	APUFI	Tranquille
Quare		597	614	546
Cumaná [24]	**235**		499	438
APUFI	**236**	**205**		469
Tranquille	**202**	**164**	**182**	

Numbers above the diagonal refer to the total number of polymorphisms in the dataset, numbers in bold refer to polymorphisms representing one distinct EST each.

**Figure 2:**
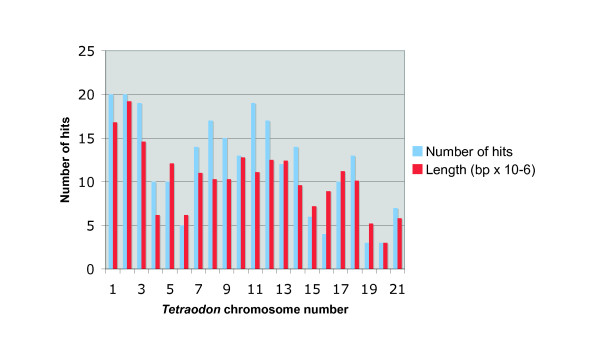
**Mapping of guppy ESTs to *Tetraodon nigroviridis *chromosomes**. ESTs for which polymorphic markers were identified by resequencing of genomic DNA were mapped to chromosomes of *T. nigroviridis*, according to their best hits. Relative length of each *T. nigroviridis *chromosome (blue) and number of guppy markers (red) are plotted. Of 387 significant hits, 136 (38%) were assigned to the fraction of the *T. niroviridis *genomic sequences not yet annotated by chromosome number.

BLAST searches with the guppy ESTs selected for marker development were performed against the *Tetraodon nigroviridis *genome. The number of hits for each chromosome was approximately proportionate to the length of each *T. nigroviridis *chromosome and indicated that at least two markers corresponding to each *Tetraodon *chromosome had been identified. Another 38% of these sequences aligned to the 40% of the *Tetraodon *sequences that were not yet assigned to one of its 21 chromosomes (Fig. 2, and data not shown). Similarly, guppy ESTs were aligned to genomic sequences of zebrafish (*Danio rerio*), fugu (*Takifugu rubripes*) and medaka (*Oryzias latipes*), to facilitate prediction of potential coding sequences in regions of interest for mapping, genomic walking, and for prediction of exon-intron boundaries (data not shown). Since a certain degree of synteny between different fish species has been previously described [10,24], a higher marker density in regions of interest for genetic mapping may be achieved by ortholog cloning based on synteny predictions on genes in the guppy. However, even between close relatives, this approach will not work perfectly, given the fact that chromosome numbers are variable and chromosomal rearrangements occur frequently.

### Molecular phylogeny of the guppy

Previous molecular phylogenetic studies on guppies were primarily based on mitochondrial sequences [25]. The molecular resources we developed enable studies on molecular evolution of nuclear genes, e.g. the identification of genes that have rapidly diversified and are potentially under positive selection, as exemplified by a study comparing genes encoding the long wave length sensitive opsins to other opsins [26].

Additional applications include studies on molecular phylogeny of the guppy. Coding sequences of orthologous expressed nuclear genes from nine different fish species, including the guppy, were identified by reciprocal BLAST. For phylogenetic analysis, these sequences were concatenated and a BIONJ tree was reconstructed [27], using SplitsTree4 software, version 4.6 [28] [see Additional file 1]. The selected genes include highly conserved ribosomal proteins as well as metabolic enzymes and transcription factors [see Additional file 2]. The number of teleost species in this analysis was limited by available sequence data from so-called non-model species, since the intersection of orthologous genes got smaller the more non-model species were included.

The resulting topology was essentially the same when only four coding sequences were concatenated. By convergence tests using increasing numbers of nuclear genes we showed that the pairwise distance statistics and the bootstrap values further improved upon addition of more sequences. This showed that a stable tree topology requires seven or more genes (data not shown). Furthermore, the topology of the phylogenetic tree was the same when maximum likelihood or maximum parsimony estimation was used instead of BIONJ (data not shown). This is in agreement with previously published comparisons of reconstruction methods, showing that BIONJ is not inferior to maximum likelihood [29]. This tree confirms previous phylogenetic studies on Poeciliid fishes, based on morphological criteria [30,31] and on genomic sequences [25,32,33]. In the future, intraspecific polymorphisms found in nuclear genes of feral guppy populations of different origins will be compared to previous phylogenies of different guppy strains [34] (Willing et al. in preparation). This will enable investigation of population structure and mapping of quantitative adaptive traits.

## Conclusion

We established a non-normalized EST library of the guppy from embryos and six different adult tissues, containing 18,000 entries with three-fold redundancy on average. We show that re-sequencing of 3' UTRs from genomic DNA of different guppy strains is a powerful strategy to identify polymorphisms existing between feral guppies of geographically different origins.

Sequence information of 10 nuclear coding genes is sufficient to reconstruct a robust teleost phylogeny including the guppy *Poecilia reticulata*.

## Methods

### Guppy Strains

We used the laboratory strains Istanbul wild and Blue [15] as well as offspring from wild-caught guppies that had been kept in the laboratory for multiple generations. The wild guppies were from the following locations: Lower Oropuche and Quare Rivers strains (Oropuche Drainage, Trinidad); Tranquille and APUFI strains (Caroni Drainage, Trinidad); Cumaná guppies (central Cumuná, Ve; EnCCFR) [35], and PV6 (Rio San Miquel, Estado Sucre, Ve).

### RNA isolation

Whole late-eyed and very late-eyed embryos were isolated from euthanised pregnant females. Embryos as well as newborn fish and tissues from adult guppies were frozen in liquid nitrogen. Frozen embryos, newborn fish, or small pieces of tissue (Table 1) were homogenized for 90 seconds using a rotor-stator homogenizer (Polytron Pt 1200, Kinematica) at full speed and total RNA was isolated using an RNAeasy Miniprep or Midiprep kit (Qiagen). RNA from male skin was prepared using Trizol (Invitrogen). Starting with total RNA extracted from whole embryos of the Quare6 and Tranquille strains, newborn fish (Tranquille), adult liver (Tranquille), testis (Blue), or skin (Oropuche2), polyA+ RNA was enriched using Quiagen Oligotex on a spin column.

### cDNA Libraries

Total RNA was reverse transcribed and cloned into a pDNRlib vector utilizing a creatorSMART™ cDNA construction kit (Clonetech). The cDNA was amplified by 18 cycles of long distance PCR using BD Advantage 2PCR™ enzyme mixture, following the manufacturer's protocols.

Alternatively, cDNA was transcribed from polyA+ RNA using the manufacturer's protocol for primer extension, which included only 6 to 10 cycles of PCR. Size fractionation of the double stranded cDNA after digestion with SfiI was performed on a 1.2% low melting agarose gel stained for 2 min with 0.1% methylene blue. After ligation, the products were dialysed against H_2_O for 20 minutes on a 0.025 μm Millipore filter before transformation into electro competent ElectroTen-Blue cells (Stratagene). Clones were picked at random and plasmid DNA was isolated with a BioRobot 8000 using MagAttract 96 minipreps (Qiagen). cDNAs were sequenced from both ends using the pDNRlib-specific vector primers pDNRfw: AGTCAGTGAGCGAGGAAGC or pDNRrev: CCAAACGAATGGTCTAGAAAG on an Applied Biosystems 3730xl DNA Analyzer. The resulting sequence trace files in ABI format were processed with pregap4 (Staden package), using the phred base calling algorithm [36]. Vector sequence and low quality sequence was trimmed [21].

The cDNA was preliminarily annotated based on the best hit resulting from BLAST (version 2.5.15) or BLASTX against the actual version of vertebrate databases available in the GCG Wisconsin Package (em_vrt, embl_new, tags_new, nr) [22].

### EST database

A Perl script ran nightly NCBI BLASTN and BLASTX jobs for new ESTs as well as for ESTs with BLAST results older than a given time period. The BLAST information, including clone name, strain and tissue of origin, were stored in the MySQL EST database (MySQL version 5.027-max; binaries and documentation can be downloaded from [14]. A PHP (version 5.1.2) web interface allows user-friendly access to this information and download of all sequences in FASTA format.

Additional links allow direct access to alignments of guppy ESTs with genomic sequences of *Oryzias latipes*, *Tetraodon nigroviridis*, and *Takifugu rubripes *[37]16,200 ESTs with a minimum length of 200 bp available sequence have been submitted to Genbank (see Table 1 for accession numbers).

The internal features of the database include a PHP web interface for administrative tasks like uploading new ESTs, updating of annotation, adding and changing position of introns (predicted and found in genomic sequences) as well as entering links to markers listed in the MySQL marker database that also has a PHP web interface.

### Analysis of genomic sequences

Gene-specific PCR primers (18 to 23 mers, T_m _59° to 62°C) were designed from *P. reticulata *cDNAs using the Primer3 program (release 1.0) [38]. Genomic DNA was extracted using a DNeasy Tissue kit (Qiagen), and was amplified by PCR using a mixture of Taq polymerase and Pfu (Fermentas; 1000:1 dilution) and following the protocol: 94°C for 5 min; 5 cycles of 94°C; 30 sec; 60°C, 30 sec; 68°C, 90 sec, followed by 31 cycles of 94°C, 30 sec; touchdown to annealing temperature from 68 to 56°C, 30 sec; 68°C, 90 sec, and a final elongation step of 68°C for 6 min. PCR products were purified using Bioline Quick Clean and sequenced, using the same forward primers as in the original PCR. Alternatively, M13forward and reverse tags had been added to the gene-specific primers; the PCR protocol was 94°C for 5 min, 35 cycles of 94°C, 30 sec; 59°C, 30 sec; 68°C, 90 sec; 68°C for 6 min. Sequencing was performed using M13 forward (5'-TGTAAAACGACGGCCAGT-3') or reverse (5'-CAGGAAACAGCTATGACC-3') primers.

### Phylogenetic analysis

Genes were included in the dataset if they met the following criteria: (1) orthologous sequences in each of ten species, and (2) alignable over most of the coding region. Genes were searched by reverse BLAST between the guppy and each of the nine other species, and considered to be homologues if they had significant TBLASTX scores (E-value < 10^-50^) and reliable sequence identity values (minimum 70% identity with *D. rerio *and more than 95% identity with *X. maculatus*). The open reading frame of each gene was manually examined after translating the nucleotide sequences with the *getorf *module from the EMBOSS package [39], version 3.0 [40].

The coding regions were aligned in a codon-based manner, by translating the nucleotide sequences into peptide sequences with *transeq *included in the EMBOSS package and than aligning them with MUSCLE, version 3.6 [41]. Afterwards the nucleotide alignment was done based on the peptide alignment using the *tranalign *module included in the EMBOSS package [39]. Flanking gap regions were deleted.

For tree estimation, the ten nucleotide alignments for each species were concatenated to obtain a single alignment. The tree shown in Additional file 1 was reconstructed using BIONJ [26] implemented in Splitstree4 version 4.6 [28].

## Note added in proof

The paper by Warthmann, Fitz and Weigel, submitted has now been accepted for publication:      Warthmann, N., Fitz, J., and Weigel, D. (2007) MSQT for choosing SNP assays from multiple DNA alignments. Bioinformatics, accepted for publication.

## Authors' contributions

CD constructed EST libraries, supervised the project, and wrote the paper, MH isolated and amplified genomic DNA, and identified the polymorphisms, MH and CL shared the sequencing projects, EMW did the phylogenetic reconstruction, MR and AS designed and maintained the MySQL databases, AS and CD assigned GO criteria, NW designed the primers, developed the MSQT and processed the sequences for SNP assay development, NT contributed to EST analysis and primer design, SRH supervised EMW, MR, and AS, provided bioinformatics tools and designed the MySQL database scheme, DW initiated the guppy project, co-supervised it and revised the manuscript.

All authors read and approved the final manuscript.

## Supplementary Material

Additional file 1 Phylogenetic reconstruction based on ten concatenated nuclear genes. This figure demonstrates application of expressed nuclear genes of the guppy for phylogenetic reconstruction. Click here for file

Additional file 2 Accession numbers of nuclear genes used for teleost phylogeny including guppy. Table of accession numbers of sequences used for phylogenetic reconstruction shown in additional file 1. Click here for file

## References

[B1] Winge Ö (1927). The location of eighteen genes in *Lebistes reticulatus *. J Genetics.

[B2] Endler JA (1991). Variation in the appearance of guppy color patterns to guppies and their predators under different visual conditions. Vision Res.

[B3] Endler JA (1995). Multiple-trait coevolution and environmental gradients in guppies. Trends Ecol Evol.

[B4] Traut W, Winking H (2001). Meiotic chromosomes and stages of sex chromosome evolution in fish: zebrafish, platyfish and guppy. Chromosome Res.

[B5] Winge Ö, Ditlevsen E (1947). Colour inheritance and sex determination in Lebistes. Heredity.

[B6] Khoo G, Lim MH, Suresh H, Gan DK, Lim KF, Chen F, Chan WK, Lim TM, Phang VP (2003). Genetic linkage maps of the guppy (Poecilia reticulata): assignment of RAPD markers to multipoint linkage groups. Mar Biotechnol (NY).

[B7] Watanabe T, Nakajima M, Yoshida M, Taniguchi N (2004). Construction of six linkage groups in the guppy (Poecilia reticulata). Anim Genet.

[B8] Watanabe T, Yoshida M, Nakajima M, Taniguchi N (2005). Linkage mapping of AFLP and microsatellite DNA markers with the body color- and sex- determining loci in the guppy (Poecilia reticulata). Zoolog Sci.

[B9] Kazianis S, Nairn RS, Walter RB, Johnston DA, Kumar J, Trono D (2004). The genetic map of Xiphophorus fish represented by 24 multipoint linkage groups. Zebrafish.

[B10] Brummell M, Kazianis S, Davidson WS, Breden F (2006). Conservation of Synteny Between Guppy and Xiphophorus Genomes. Zebrafish.

[B11] Shimizu N, Sasaki T, Asakawa S, Shimizu A, Ishikawa SK, Imai S, Murayama Y, Himmelbauer H, Mitani H, Furutani-Seiki M (2006). Comparative genomics of medaka and fugu. Comparative Biochemistry and Physiology, Part D.

[B12] Magurran AE (2005). Evolutionary Ecology: The Trinidadian Guppy.

[B13] Altschul SF, Gish W, Miller W, Myers EW, Lipman DJ (1990). Basic local alignment search tool. Molecular Biology and Evolution.

[B14] MySQL. http://www.mysql.org.

[B15] Dzwillo M (1959). Genetische Untersuchungen an domestizierten Stämmen von Lebistes reticulatus (Peters). Mitteilungen des Hamburgischen Zool Museum Inst.

[B16] Ashburner M, Ball CA, Blake JA, Botstein D, Butler H, Cherry JM, Davis AP, Dolinski K, Dwight SS, Eppig JT (2000). Gene ontology: tool for the unification of biology. The Gene Ontology Consortium. Nat Genet.

[B17] Paschall JE, Oleksiak MF, VanWye JD, Roach JL, Whitehead JA, Wyckoff GJ, Kolell KJ, Crawford DL (2004). FunnyBase: a systems level functional annotation of Fundulus ESTs for the analysis of gene expression. BMC Genomics.

[B18] Pearson WR, Lipman DJ (1988). Improved tools for biological sequence comparison. Proc Natl Acad Sci USA.

[B19] Mazumder B, Seshadri V, Fox PL (2003). Translational control by the 3'-UTR: the ends specify the means. Trends Biochem Sci.

[B20] Jurinke C, van den Boom D, Cantor CR, Koster H (2001). Automated genotyping using the DNA MassArray technology. Methods Mol Biol.

[B21] Staden R, Beal KF, Bonfield JK (2000). The Staden package, 1998. Methods Mol Biol.

[B22] Accelrys GCG 11.0 GenHelp Online Documentation. http://www.accelrys.com/support/bio/genhelp/.

[B23] Becher SA, Russell ST, Magurran AE (2002). Isolation and characterization of polymorphic microsatellites in the Trinidadian guppy (*Poecilia reticulata*). Molecular Ecology Notes.

[B24] Xu P, Wang S, Liu L, Peatman E, Somridhivej B, Thimmapuram J, Gong G, Liu Z (2006). Channel catfish BAC-end sequences for marker development and assessment of syntenic conservation with other fish species. Animal Genetics.

[B25] Breden F, Ptacek MB, Rashed M, Taphorn D, Figueiredo CA (1999). Molecular phylogeny of the Live-Bearing Fish Genus *Poecilia *(Cyprinodontiformes: Poecilidae). Molecular Phylogenetics and Evolution.

[B26] Hoffmann M, Tripathi N, Henz SR, Lindholm AK, Weigel D, Breden F, Dreyer C (2007). Opsin gene duplication and diversification in the guppy, a model for sexual selection. Proc Biol Sci.

[B27] Gascuel O (1997). BIONJ: an improved version of the NJ algorithm based on a simple model of sequence data. Mol Biol Evol.

[B28] Huson DH, Bryant D (2006). Application of phylogenetic networks in evolutionary studies. Mol Biol Evol.

[B29] Guindon S, Gascuel O (2002). Efficient biased estimation of evolutionary distances when substitution rates vary across sites. Mol Biol Evol.

[B30] Rosen DE, Bailey RM (1963). The poeciliid fishes (Cyprinodontiformes), their structure, zoogeography and stystematics. Bull Am Mus Nat Hist.

[B31] Parenti LR, Rauchenberger M, Meffe GK, Snelson FF (1989). Systematic overview of the Poeciliines. Ecology and evolution of livebearing fishes (Poeciliidae).

[B32] Volff JN, Korting C, Meyer A, Schartl M (2001). Evolution and discontinuous distribution of Rex3 retrotransposons in fish. Mol Biol Evol.

[B33] Hrbek T, Seckinger J, Meyer A (2007). A phylogenetic and biogeographic perspective on the evolution of poeciliid fishes. Mol Phylogenet Evol.

[B34] Fajen A, Breden F (1992). Mitochondrial DNA sequence variation among natural populations of the Trinidad Guppy. Evolution.

[B35] Alexander HJ, Breden F (2004). Sexual isolation and extreme morphological divergence in the Cumana guppy: a possible case of incipient speciation. J Evol Biol.

[B36] Ewing B, Hillier L, Wendl M, P G (1998). Basecalling of automated sequencer traces using phred. I. Accuracy assessment. Genome Research.

[B37] Guppy Databases. http://guppy.weigelworld.org.

[B38] Rozen S, Skaletsky HJ, Krawetz S, Misener S, Totowa, NJ (2000). Primer3 on the WWW for general users and for biologist programmers. Bioinformatics Methods and Protocols: Methods in Molecular Biology.

[B39] EMBOSS. http://emboss.sourceforge.net/apps.

[B40] Rice P, Longden I, Bleasby A (2000). EMBOSS: the European Molecular Biology Open Software Suite. Trends Genet.

[B41] Edgar RC (2004). MUSCLE: multiple sequence alignment with high accuracy and high throughput. Nucleic Acids Res.

