# Supplementary material for: ESTs and EST-linked polymorphisms for genetic mapping and phylogenetic reconstruction in the guppy, Poecilia reticulata
Source: BMC Genomics. 2007 Aug 8;8:269. doi: 10.1186/1471-2164-8-269 (PMC1994688; doi:10.1186/1471-2164-8-269)
Supplement: Additional file 1 — Phylogenetic reconstruction based on ten concatenated nuclear genes. This figure demonstrates application of expressed nuclear genes of the guppy for phylogenetic reconstruction. [file 1471-2164-8-269-S1.pdf]

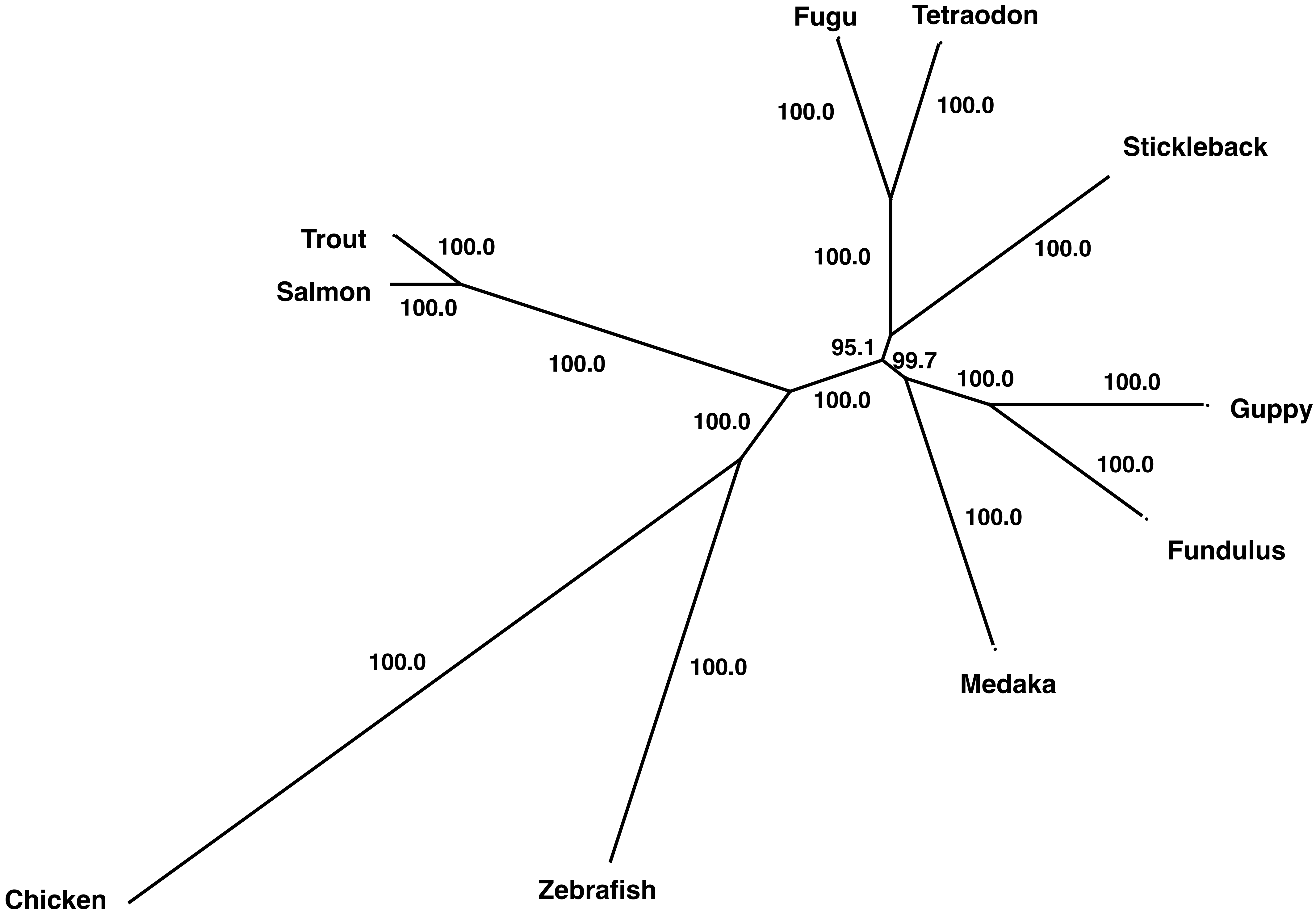

Fugu
Tetraodon
100.0
100.0
Stickleback
100.0
100.0
Trout
100.0
Salmon
100.0
100.0
95.1
99.7
100.0
100.0
100.0
Guppy
100.0
100.0
Fundulus
100.0
Medaka
100.0
100.0
Chicken
Zebrafish

Phylogenetic reconstruction based on ten concatenated nuclear genes.

Coding sequences of the 10 nuclear genes listed in Table S1 were concatenated for tree reconstruction. Of 5801 total characters used, 833 variable characters were parsimony-uninformative, and 1467 were parsimony-informative. Reliability was assessed using bootstrap re-sampling of 20,000 replicates, and bootstrap values are indicated. The species are *Fundulus heteroclitus* (Fundulus), *Oryza latipes* (Medaka), *Gasterosteus aculeatus* (Stickleback), *Tetraodon nigroviridis* (Tetraodon), *Fugu rubipes* (Fugu), *Oncorhynchus mykiss* (Trout), *Salmo salar* (Salmon), *Danio rerio* (Zebrafish), *Poecilia reticulata* (Guppy). The outgroup is the chicken, *Gallus gallus* (Gallus).
