# Supplementary material for: ESTs and EST-linked polymorphisms for genetic mapping and phylogenetic reconstruction in the guppy, Poecilia reticulata
Source: BMC Genomics. 2007 Aug 8;8:269. doi: 10.1186/1471-2164-8-269 (PMC1994688; doi:10.1186/1471-2164-8-269)
Supplement: Additional file 2 — Accession numbers of nuclear genes used for teleost phylogeny including guppy. Table of accession numbers of sequences used for phylogenetic reconstruction shown in additional file 1. [file 1471-2164-8-269-S2.pdf]

Additional Table 1. Accession numbers* of nuclear genes used for Teleost phylogeny including guppy

	Species									
Gene	Fundulus	Medaka	Gasterosteus	Tetraodon	Fugu	Trout	Salmon	Gallus	Zebrafish	Guppy (cDNA/Acc.Nr.)
EF 1 alpha	AY430091.1<br>GenBank	AB013606.1<br>GenBank	CD506955.1<br>GenBank	GSCT<br>00011211001	GENSCANSL<br>ICE<br>00000025408	CF752834.1<br>GenBank	AF321836.1<br>GenBank	NM_204157.2<br>GenBank	ENSDART<br>00000023156	Oro_Skin_3_I03<br>GenBank: EF408829
cyclin G1	DN957128.1<br>GenBank	BJ885147.1<br>GenBank	DT969609.1<br>GenBank	GSTENT<br>00017018001	NEWSINFRU<br>T<br>00000173819	BX870371.3<br>GenBank	AM083796.1<br>GenBank	BX935990.2<br>GenBank	AY423016.1<br>GenBank	Tra_Embryo_14_L05<br>GenBank: EF408837
Heat Shock<br>Cognate	CV819660.1<br>GenBank	BJ715323.1<br>GenBank	DW622253.1<br>GenBank	CR699773.2<br>GenBank	NEWSINFRU<br>T<br>00000149686	CF752396.1<br>GenBank	DY703130.1<br>GenBank	AJ004940.1<br>GenBank	ENSDART<br>00000063864	Qua_Embryo_U5_O15<br>GenBank: EF408831
Lactate Dehydro-<br>genase B	M33969.1<br>GenBank	BJ728879.1<br>GenBank	DN717250.1<br>GenBank	GWSHT<br>00003443001	NEWSINFRU<br>T<br>00000160007	BX079778.2<br>GenBank	DW581686.1<br>GenBank	NM_204177.1<br>GenBank	ENSDART<br>00000010777	Blu_Testis_3_H19<br>GenBank: EF408825
phosphoglycerate<br>Kinase	CN985009.1<br>GenBank	AB111386.1<br>GenBank	DN680833.1<br>GenBank	CR682765.2<br>GenBank	NEWSINFRU<br>T<br>00000160355	CA356380.1<br>GenBank	DY709316.1<br>GenBank	NM_204985.1<br>GenBank	ENSDART<br>00000076333	Blu_Testis_7_N09<br>GenBank: EF408827
ribosomal P. L27	CN981470.1<br>GenBank	AV669486.1<br>GenBank	DW032853.1<br>GenBank	GSTENT<br>00021244001	NEWSINFRU<br>T<br>00000137482	CA341841.1<br>GenBank	BU965625.1<br>GenBank	NM_205337.1<br>GenBank	ENSDART<br>00000020311	Tra_Embryo_4-3_A01<br>GenBank: EF408835
ribosomal P. L7	CN978636.1<br>GenBank	BJ895217.1<br>GenBank	DN729533.1<br>GenBank	CR680002.2<br>GenBank	NEWSINFRU<br>T<br>00000133890	CA385201.1<br>GenBank	DW536733.1<br>GenBank	NM_001006345.<br>1<br>GenBank	ENSDART<br>00000019149	Is_Skin_2_G06<br>GenBank: EF408834
ribosomal P. S15	CN991454.1<br>GenBank	BJ495529.1<br>GenBank	DN672832.1<br>GenBank	GSTENT<br>00020904001	NEWSINFRU<br>T<br>00000146235	CA347051.1<br>GenBank	DW539978.1<br>GenBank	NM_205462.1<br>GenBank	ENSDART<br>00000066146	Oro_Skin_5_B04<br>GenBank: EF408830
ribosomal P. S6	CN980370.1<br>GenBank	BJ016476.1<br>GenBank	CD493077.1<br>GenBank	CR661743.2<br>GenBank	NEWSINFRU<br>T<br>00000141607	CA365709.1<br>GenBank	CA057133.1<br>GenBank	NM_205225.1<br>GenBank	FGENESH<br>00000072054	Oro_Retina_2_H03<br>GenBank: EF408828
triosephosphate<br>Isomerase B	CV816996.1<br>GenBank	BJ727505.1<br>GenBank	DN654949.1<br>GenBank	GSTENT<br>00005289001	NEWSINFRU<br>T<br>00000156180	CF752393.1<br>GenBank	DW566697.1<br>GenBank	NM_205451.1<br>GenBank	ENSDART<br>00000060056	Qua_Embro_6-3_H08<br>GenBank: EF408833

Species listed in the first line are as explained in legend of additional Figure 1.

* Accession numbers refer to GenBank where indicated, all others are from the Ensembl Genome Browser [http://www.ensembl.org/].
